# Feasibility of Endoscopic Submucosal Dissection: A New Technique for En Bloc Resection of a Large Superficial Tumor in the Colon and Rectum

**DOI:** 10.1155/2011/948293

**Published:** 2011-05-02

**Authors:** T. Shono, K. Ishikawa, Y. Ochiai, M. Nakao, O. Togawa, M. Nishimura, S. Arai, K. Nonaka, Y. Sasaki, H. Kita

**Affiliations:** ^1^Department of Gastroenterology, International Medical Center, Saitama Medical University, 1397-1 Yamane, Hidaka, Saitama 350-1298, Japan; ^2^Department of Gastroenterology and Hepatology, Graduate School of Medical Sciences, Kumamoto University, 1-1-1 Honjo, Kumamoto 860-8556, Japan

## Abstract

Endoscopic submucosal dissection (ESD) is a promising procedure that enables en bloc resection of large superficial tumors in the upper gastrointestinal tract. On the other hand, ESD in the colon and rectum is technically difficult to perform because of its anatomical features. At our institution, 137 consecutive superficial colorectal tumors larger than 20 mm in diameter in 137 patients were treated by ESD between April 2007 and October 2010, and 132 lesions were successfully resected. The average procedure time was 79.2 minutes, and the rate of en bloc resection was 89.1% (122/137). The rate of complete resection, defined as en bloc resection with tumor-free lateral and vertical margins, was 85.4% (117/137). The rate of perforation was 3.6% (5/137). Colorectal ESD achieved a high rate of en bloc resection and complete resection and is applicable in the colorectum.

## 1. Introduction

Colorectal neoplastic lesions sometimes extend laterally rather than vertically, and colorectal tumor that spreads superficially was named laterally spreading tumor (LST) by Kudo et al. [1]. Theoretically, colorectal superficial neoplastic lesions, including LST, without lymph node metastasis can be treated by intraluminal endoscopic resection alone. Endoscopic mucosal resection (EMR) is widely used as an established method for colorectal superficial neoplasms although the indication for EMR has been limited by the snare size. Endoscopic piecemeal mucosal resection (EPMR) and laparoscopic resection have been accepted as treatments for large superficial tumors over 20 mm in the colon.

Endoscopic submucosal dissection (ESD) is widely accepted as a reliable method that enables en bloc resection of large superficial tumors in the upper gastrointestinal (GI) tract, including the esophagus and stomach, in Japan. By contrast, ESD for the colorectal neoplasm has been regarded as a technically difficult therapeutic procedure because of the anatomical and histological differences between the upper GI and the colorectum. Recently, with the development of related equipment, colorectal ESD has gradually been carried out in Japanese institutions with favorable results [2–10]. We report the feasibility of this technique using our own results. 

## 2. Materials and Methods

### 2.1. Patients and Indication for Colorectal ESD

Between April 2007 and October 2010, 137 consecutive colorectal superficial tumors in 137 patients (79 men, 58 women; mean age 66.8 years), which fulfilled our indication criteria, were treated with ESD technique, at Saitama Medical University, International Medical Center. The colorectal ESD standardization implementation working group suggested the indication for colorectal ESD, as shown in Table 3 [10], and our study was carried out based on the same criteria. In brief, the indications for ESD were colorectal superficial neoplasia that might have a malignant component without lymph node metastases larger than 20 mm in diameter or that are difficult to completely remove with the conventional EMR technique. The indication for colorectal ESD was decided from the endoscopic features, observed with chromoendoscopy and magnifying endoscopy with narrow band imaging (NBI) in order to recognize the demarcated line between normal mucosa and lesion and to estimate the depth of the lesion. Endoscopic ultrasonography (EUS) was also performed when conventional endoscopy raised the suspicion of massive submucosal invasion. Written informed consent was obtained from all patients. ESD was performed by four endoscopists, and the therapeutic efficacy and safety were assessed. 

### 2.2. Technical Method of Colorectal ESD

**Figure 1:**
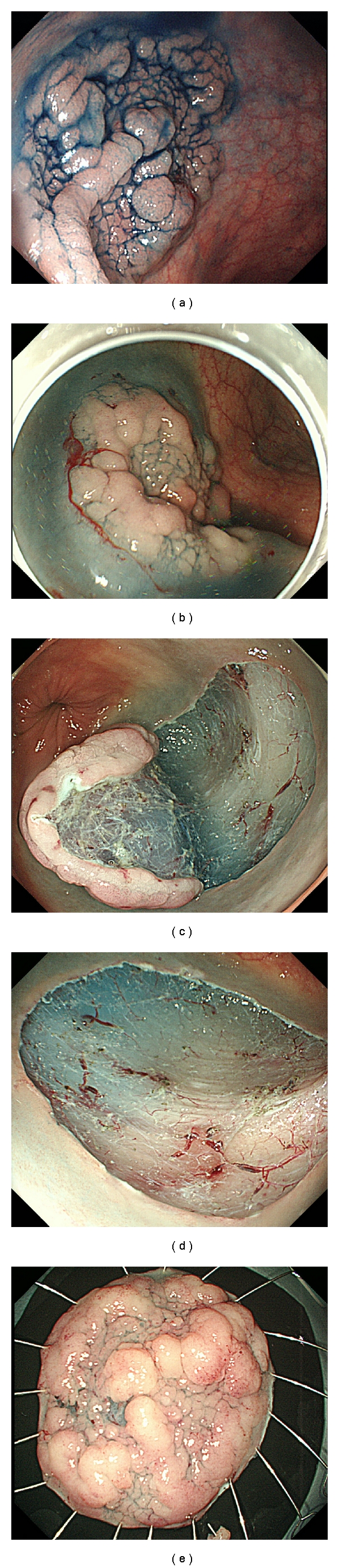
(a) Conventional endoscopic view with indigo carmine dye spray revealed laterally spreading tumor (LST), 40 mm in diameter, located in the rectum. The border was well demarcated. (b) After injection of sodium hyaluronate. (c) Mucosal incision and dissection. (d) Rectal ulcer after ESD. (e) Resected specimen was 45 mm × 40 mm.

The ESD procedure has been described elsewhere [11–13]. That typical procedure of colorectal ESD is shown in Figure 1. ESD was performed using two types of colonoscopes (PCF-Q260AI, PCF-Q240ZI; Olympus Medical Systems, Tokyo, Japan). A tip-transparent hood was attached to the tip of the endoscope. A high-frequency power supply VIO300D (ERBE Elektromedizin, Germany) was used. A flush knife (DK2618JN15; Fijinon, Tokyo, Japan) was used in 100 cases. A hook knife (KD-620LR: Olympus, Tokyo, Japan) was used in combination with the flush knife in 7 patients with difficult dissection. A precutting knife (KD-10Q-1; Olympus) was used in 37 cases because the flush knife was not available when we started this study. We then gradually switched to the flush knife during this study because it was developed specifically for ESD. Carbon dioxide insufflation described by Saito et al. [14] was used during colorectal ESD in order to reduce the patient's discomfort during and after ESD. Conscious sedation was achieved with a small amount of midazolam and pethidine.

Normal saline was preinjected to avoid injecting sodium hyaluronate into the wrong layer because it is difficult to inject sodium hyaluronate solution into the appropriate submucosal layer of the colon. Sodium hyaluronate is useful [15] and necessary for colorectal ESD. Sodium hyaluronate 0.4% (Mucoup; Johnson & Johnson, Tokyo, Japan) mixed with a small amount of indigo carmine dye and epinephrine was used as the liquid for local injection into submucosal layer. By mixing a small amount of indigo carmine dye and epinephrine into sodium hyaluronate solution, visualization of the submucosal layer to be dissected was much easier and it diminished bleeding during the procedure.

The range of the colonic neoplasm was generally clear and marking was not necessary. In most cases, mucosal incision started from the most distant edge to determine the end line to be dissected. To take advantage of gravity, changing the patient's position was important. When the relevant dissection layer was sufficiently visible, circumferential incision was performed. Hemostatic forceps (SDB2422; Pentax Hoya) were used to control bleeding. Any visible large vessels were precoagulated and cut. The resected specimen was removed and evaluated histopathologically. 

### 2.3. Followup

Colonoscopy was scheduled at 6 and 12 months after ESD, and then annually. Suspicious abnormalities were histologically confirmed by biopsy.

## 3. Results

The clinicopathologic characteristics of 137 patients are described in Table 1. The mean diameter of the lesions was 29.2 mm (range 20–150), and the mean procedure time for resection was 79.2 minutes (range 20–300). Macroscopic feature of the tumors included LST type in 100 lesions and non-LST type in 37 lesions. The lesions were located in the cecum (*n* = 10), ascending colon (*n* = 26), transverse colon (*n* = 32), descending colon (*n* = 5), sigmoid colon (*n* = 28), and rectum (*n* = 36). We tried to endoscopically resect 137 superficial tumors in the colon and rectum and successfully resected 132 superficial tumors. Endoscopic treatment was suspended in 5 cases because of strong submucosal fibrosis in 4 cases and procedure-related perforation in one case, as shown in Table 2. En bloc resection was performed in 122 cases, with a rate of 89.1% (122/137). The remaining ten superficial tumors were resected in two to six pieces by the combination of ESD and EMR. The rate of complete resection, defined as en bloc resection with tumor-free lateral and vertical margins, was 85.4% (117/137) because 9 cases were histologically judged to have a positive lateral margin and one case a positive vertical margin. 

Complications of the treatments included postoperative hemorrhage which required clipping in 5 (3.6%) cases. Perforations occurred during ESD in 4 cases and after ESD in one case. Two cases of perforation during ESD were subjected to surgical operation within 24 hours, including curative resection of the tumor, while the remaining 3 cases were cured conservatively after endoscopic closure using clips. The histopathological diagnoses of the resected specimens were tubular and tubulovillous adenomas in 40 cases (29.2%), adenomas with focal carcinoma or carcinomas localized in the mucosa in 89 cases (65%), and carcinomas with invasion into the submucosa in 8 cases (5.8%). In eight submucosal invasive cancers, there were three SM1 cancers and five SM2 (SM invasion 1000 *μ*m or more from muscularis mucosae), without vessel infiltration. Five SM2 cancers were considered to be at substantial risk for nodal metastasis, so laparoscopic colectomy and lymphadenectomy were additionally performed and distant metastases were not detected. In the follow-up period, there were no cases of local recurrence and no instances of metastasis.

Our study included 132 cases of newly developed lesions and five residual lesions that showed a non-lifting sign. These five residual lesions were also selected and subjected to analysis. Of these 5 cases, en bloc resection was completed in one case, piecemeal resection was performed in 2 cases, and endoscopic treatment was suspended in 2 cases. 

## 4. Discussion

Compared with conventional surgery, endoscopic treatment has the advantage of being less invasive and less costly [16]. Large superficial tumors in the colon and rectum have little tendency toward vertical growth despite their lateral extension; therefore, endoscopic resection is supposed to be preferable [11]. This means that colorectal ESD, a minimally invasive technique in colorectal cancer, is now playing an important role in the treatment of large superficial colorectal tumors.

Eight clinical studies regarding the efficiency and safety of ESD in the colon and rectum have been reported thus far between 2007 and 2010 [2–9], all from Japan. In these eight studies, the en bloc resection rate in colorectal ESD was reported to be 80–98.6%, and the complete resection rate was 70–98.6%. In our study, the rate of en bloc resection was 89.1% (122/137), and the rate of complete resection was 85.4% (117/137), similar to the previous reports. This is probably because the technique of ESD in the colon and rectum is in common with gastric ESD, and endoscopistscan experience gastric ESD procedure to some extent before they started ESD in the colon and rectum. On the other hand, the rate of perforation in the previous eight reports ranged 1.4–10.4%, while the rate of perforation in our data was 3.6%, better than the average level of these reports. Because different knives were used in each of the eight previous reports, the choice of knife may be in part related to the rate of perforation in each experience, including our results. 

**Figure 2:**
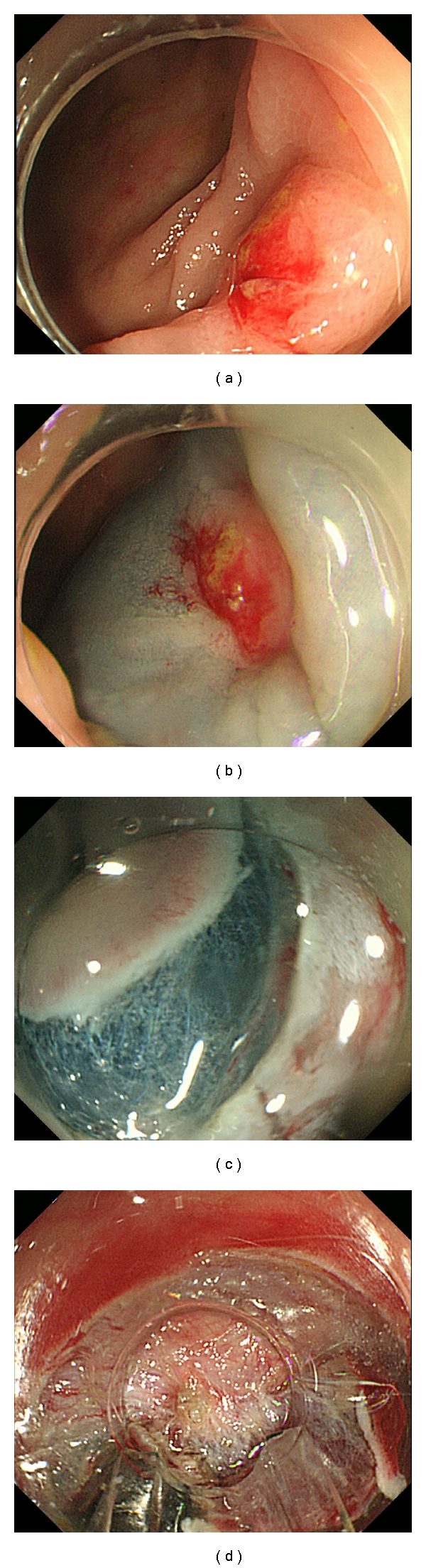
A case of ESD failure. (a) Residual mucosal lesion after EMR located in the hepatic flexure of the colon. (b) Non-lifting sign was positive after submucosal injection. (c) Submucosal layer was clearly visualized after incision. (d) Severe submucosal fibrosis existed under the lesion. ESD procedure was suspended to avoid perforation.

Among the 5 cases that failed to complete the ESD procedure, 4 were suspended due to severe fibrosis (Figure 2). It is well known that either biopsy from the lesion or a previous history of endoscopic treatment can often cause submucosal fibrosis. Indeed, two of the five suspended cases had a previous history of endoscopic treatment at the same location, ESD was attempted for recurrent lesions, and perforation also occurred in one of the five cases, in agreement with the report by Isomoto et al. [6] that the finding of fibrosis was one of the major risk factors of perforation. Magnified endoscopic observation with NBI or crystal violet staining is necessary to perform successful ESD. It allows discrimination between benign and malignant tumors and predicts the tumor infiltration depth [17–20], and precise diagnosis before ESD may lead to good strategies for ESD and can also reduce the risk of perforation in colorectal ESD. Nevertheless, it is difficult to predict submucosal fibrosis. Thus, information on the past history of biopsies or endoscopic treatment is important in each case. 

Endoscopic treatment of residual lesions, positive for a non-lifting sign due to the previous endoscopic treatment, is technically much more difficult. In our study, we attempted endoscopic resection for 5 cases. Complete resection was achieved in only one case, 2 cases were treated as piecemeal resection, and we suspended endoscopic treatment in 2 cases; therefore, the choice of ESD for residual tumor requires caution.

Minimally invasive, laparoscopically assisted surgery was first considered in 1990 for patients undergoing colectomy for cancer [21]. The first laparoscopic surgery for colorectal cancer in Japan was reported in 1992. In the early phase, many cases were indicated for early cancer. The number of operations has been increasing each year, and now even some advanced cases undergo laparoscopic surgery [22]. There have been several randomized, controlled studies to determine the value of laparoscopic resection for colorectal cancer, and their results have been reported, showing the short-term results of laparoscopic surgery to be superior and survival to be almost equivalent to open operations [21, 23]. In Japan, the Japan Society for Cancer of the Colon and Rectum (JSCCR) recommended that clinical Stage I or II cancer located in the cecum, left- or right-sided colon cancer and rectosigmoid cancer are good indications for laparoscopic surgery. On the other hand, obese patients, patients with a history of abdominal surgery, or a tumor located in the transverse colon/rectum, are considered difficult cases for laparoscopic resection. These cases can be good candidates for colorectal ESD if they fulfill the indications for colorectal ESD.

In conclusion, our study confirmed, in agreement with previous reports, that colorectal ESD is developing with favorable results. It is a feasible technique and is gradually being established as a standard procedure for the large superficial colorectal tumors.

## Figures and Tables

**Table 1:** Clinicopathologic characteristics of 137 superficial colorectal tumors.

Age, mean (range), years	66.8 (40–90)
Men, Women	79, 58
Tumor Size, mean (range), mm	29.2 (20–150)
Tumor location	*n*
Cecum	10
Ascending Colon	26
Transverse Colon	32
Descending Colon	5
Sigmoid Colon	28
Rectum	36

Tumor Morphology	*n*
Laterally Spreading Tumors (LST)	100
Non LST	37

Operation time, average (range), minutes	79.2 min. (20–100)
En bloc resection rate, %	89.1% (122/137)
Complete resection rate, %	85.4% (117/137)
Endoscopic piecemeal resection (EPMR), %	7.3% (10/137)
Suspended, %	3.6% (5/137)

Complications	
Perforation, %	3.6% (5/137)
Postoperative hemorrhage, %	3.6% (5/137)

Histopathological Diagnosis	
Adenoma	40
Adenocarcinoma	97
Intramucosal	89
Submucosal (SM) invasion	8
SM1	3
SM2	5

Complete resection: En bloc resection with tumor-free lateral and vertical margins.

SM1: submucosal invasion less than 1000 *μ*m from the muscularis mucosae.

SM2: submucosalinvasion 1000 *μ*m or more fromthe muscularis mucosae.

**Table 2:** Clinicopathologic characteristics of 5 suspended cases.

No.	Age Sex	Location	Size mm	Gross type	Depth	Diagnosis	History of biopsy/ET	NLS	Cause of suspend	Additional therapy
1.	69 M	Ascending	20	LST	SM2	Adenoca.	EMR/APC	+	Severe fibrosis	Scheduled LS
2.	77 M	Transverse	15	IIa+IIc	SM1	Adenoca.	EMR	+	Severe fibrosis	Scheduled LS
3.	68 M	Sigmoid	20	IIa	M	Adenoca.	biopsy	+	Severe fibrosis	Scheduled LS
4.	65 M	Transverse	22	LST	M	Adenoca.	biopsy	+	Severe fibrosis	Scheduled LS
5.	58 M	Sigmoid	20	IIc	M	Adenoca.	biopsy	+	Perforation	Emergency Surgery

ET: Endoscopic Therapy, NLS: Non-lifting Sign, LS: Laparoscopic Surgery, Adenoca.: Adenocarcinoma.

M: intramucosal cancer.

SM1: submucosal invasion less than 1000 *μ*m from the muscularis mucosae.

SM2: submucosalinvasion 1000 *μ*m or more fromthe muscularis mucosae.

**Table 3:** Indication of endoscopic submucosal dissection (ESD) for colorectal tumor.

(1) Lesions that were larger than 20 mm in diameter in which en bloc resection using snare EMR is difficult, although it is indicative for endoscopic treatment
(i) Non-granular LST, particularly those of the pseudo-depressed type
(ii) Lesions with Vi type pit pattern
(iii) Carcinoma with submucosal infiltration
(iv) Large depressed type lesion
(v) Large lesions with elevated type suspected to be cancer^†^
(2) Mucosal lesions with fibrosis caused by prolapse due to biopsy or peristalsis of the lesions
(3) Sporadic localized tumors in chronic inflammation such as ulcerative colitis
(4) Local residual early cancer after endoscopic resection

^†^Including granular LST that consisted of large nodules.

EMR: endoscopic mucosal resection, LST: laterally spreading tumor.
